# Defining exposure for estimating the global burden of alcohol consumption: plausibility testing of WHO methodology using ASEAN countries as a case study

**DOI:** 10.1186/s13011-026-00703-y

**Published:** 2026-01-30

**Authors:** Jürgen Rehm, Gianna Gayle H. Amul, Sawitri Assanangkornchai, Surasak Chaiyasong, Daniela Correia, Noran N. Hairi, Enjeline Hanafi, Ahmed S. Hassan, Kyaw Ko Ko Htet, Wah Yun Low, John Robert C. Medina, Jiraluck Nontarak, Sok King Ong, Pol Rovira, Kristiana Siste, Bundit Sornpaisarn, Vanphanom Sychareun, Wen Ting Tong, Polathep Vichitkunakorn, Siyan Yi, Nyi Nyi Zayar, Kevin Shield

**Affiliations:** 1 Institute for Mental Health Policy Research, Centre for Addiction and Mental Health (CAMH), 250 College Street, Toronto, M5T 1R8 Ontario Canada; 2 Campbell Family Mental Health Research Institute, Centre for Addiction and Mental Health (CAMH), 250 College Street, Toronto, M5T 1R8 Ontario Canada; 3 PAHO/WHO Collaborating Centre at CAMH, 250 College Street, Toronto, M5T 1R8 Ontario Canada; 4 Dalla Lana School of Public Health, University of Toronto (UofT), 155 College St Room 500, Toronto, M5T 3M7 Ontario Canada; 5 Institute of Medical Science, Temerty Faculty of Medicine, C. David Naylor Building, 6 Queen’s Park Crescent, Suite 119, Toronto, M5S 3H2 Ontario Canada; 6 Department of Psychiatry, Faculty of Medicine, University of Toronto, 250 College Street, 8th Floor, Toronto, M5T 1R8 Ontario Canada; 7 Centre of Interdisciplinary Addiction Research (ZIS), Department of Psychiatry and Psychotherapy, University Medical Center Hamburg-Eppendorf (UKE), Martinistraße 52, 20246 Hamburg, Germany; 8 Program on Substance Abuse & WHO European Region Collaborating Centre at Public Health Agency of Catalonia, Aragó Street 330, Barcelona, Catalonia, 08009 Spain; 9 Ateneo School of Government, University Katipunan Avenue, Pacifico Ortiz Hall, Fr. Arrupe Road, Social Development Complex, Ateneo de Manila, Quezon City, Loyola Heights 1108 Philippines; 10 FORUT (Norway), Roald Amundsens veg 1B, Gjøvik, 2816 Norway; 11 Centre for Alcohol Studies, Prince of Songkla University, 15 Kanjanavanich Rd, Hat Yai, Songkhla, 90110 Thailand; 12 Social Pharmacy & Alcohol and Health Promotion Policy Research Units, Faculty of Pharmacy, Mahasarakham University, Maha Sarakham, 44150 Thailand; 13 WHO Regional Office for Europe, UN City, Marmorvej 51, Copenhagen, 2100 Denmark; 14 EPIUnit - Instituto de Saúde Pública, Universidade do Porto, Rua das Taipas 135, Porto, 4050-600 Portugal; 15 Laboratório para a Investigação Integrativa e Translacional em Saúde Populacional (ITR), Rua das Taipas 135, Porto, 4050-600 Portugal; 16 Department of Social and Preventive Medicine, Faculty of Medicine, Universiti Malaya, Kuala Lumpur, 50603 Malaysia; 17 Department of Psychiatry, Faculty of Medicine, Universitas Indonesia – dr. Cipto Mangunkusumo General Hospital, Jakarta, 10430 Indonesia; 18 Institute of Clinical Epidemiology, National Institutes of Health, University of the Philippines Manila, 623 Pedro Gil St, Ermita, Manila, 1000 Philippines; 19 Department of Epidemiology, Faculty of Public Health, Mahidol University, 420/1 Rajvithi road, Rajthevee, Bangkok, 10400 Thailand; 20 PAPRSB Institute of Health Sciences, Universiti Brunei Darussalam, Jalan Tungku Link, Gadong, Darussalam, BE1410 Brunei; 21 Department of Health Services & Department of Policy & Planning, Ministry of Health, Commonwealth Drive, Bandar Seri Begawan, BB3913 Brunei Darussalam; 22 JC School of Public Health and Primary Care, Chinese University of Hong Kong, School of Public Health Building, Prince of Wales Hospital, Shatin, New Territories Hong Kong SAR; 23 Faculty of Public Health, University of Health Sciences, Vientiane, Lao PDR; 24 Department of Primary Care Medicine, Faculty of Medicine, Universiti Malaya, Kuala Lumpur, 50603 Wilayah Persekutuan Kuala Lumpur Malaysia; 25 Department of Family and Preventive Medicine, Faculty of Medicine, Prince of Songkla University, 15 Kanjanavanich Rd, Hat Yai, Songkhla, 90110 Thailand; 26 Health Policy Research Center, Faculty of Medicine, Prince of Songkla University, 15 Kanjanavanich Rd, Hat Yai, Songkhla, 90110 Thailand; 27 Saw Swee Hock School of Public Health, National University of Singapore and National University Health System, Singapore, 117549 Singapore; 28 KHANA Centre for Population Health Research, Phnom Penh, 12301 Cambodia; 29 Department of Epidemiology and Biostatistics, Schulich School of Medicine and Dentistry, Western University, 1465 Richmond Street, London, N6G 2M1 Ontario Canada

**Keywords:** Alcoho, Alcohol *per capita* consumption, Abstention, Heavy episodic drinking, Population, Monitoring, Risk factor, ASEAN

## Abstract

**Background:**

Comparative risk assessments (CRAs) provide important information for shaping alcohol control policies. Underlying their CRAs, the WHO uses a standardised methodology to assess and detail the levels of alcohol use for all countries and for various regions. This publication uses a case study approach on the member states of the Association of South East Asian Nations (ASEAN) to examine potential biases resulting from the methodology employed by the WHO in calculating exposure values for their CRAs.

**Methods:**

Researchers from each of the 10 ASEAN member states identified large population surveys to improve upon the data collected by the WHO monitoring systems to estimate exposure between 2000 and 2022. From these surveys and aggregate data, key indicators were created for each Member State using WHO standardised methodology. Steps were defined to test for implausible values, particularly for the indicator for average level of alcohol consumption among drinkers. Sensitivity analyses were undertaken to identify possible causes of these values. Finally, we compared the results of the implausibility checks with two other regions, the European Union (EU) and the East African Community (EAC), based on data collected by the WHO.

**Results:**

The indicator for average volume of alcohol consumption among drinkers showed implausibly high values for three ASEAN countries, Lao PDR, Thailand and Viet Nam. Further simulations based on assumptions regarding the prevalence of people with heavy or very heavy drinking levels further corroborated a likely bias. An examination of the constituents of the indicator revealed that the bias for Thailand could be due to responses received to survey questions regarding alcohol abstention, in which a high number of respondents claimed no consumption of alcohol over the past year. For the Lao PDR and Viet Nam, the same problem with survey respondent self-reports on alcohol consumption may exist, but we cannot exclude the possibility that answers to the survey question regarding unrecorded alcohol may also have contributed. Investigations of two further regions of the world also showed some implausible values, albeit to a smaller degree for the EU.

**Conclusions:**

Plausibility testing of key monitoring indicators is important and yields important information for improving future monitoring efforts.

## Background

In comparative risk assessments (CRAs), alcohol consumption has been consistently identified by both the Global Burden of Disease (GBD) studies (latest iterations: [1, 2]) and by the World Health Organization (WHO) (latest iteration: [3]) as a major risk factor for global burden of disease and injuries (for a review of earlier iterations, see [4]). However, the underlying methodology between the two traditions varies considerably (for comparisons, see [5, 6]. While both groups use an attributable-fraction framework in the tradition of Levin ([7]; for principles [8]), exposure is defined differently.

GBD uses one dimension—high alcohol consumption—defined as alcohol consumption in excess of an age and regionally dependent theoretical minimum risk exposure level, i.e., the level of alcohol consumption at which all-cause risk of disability adjusted life years lost (DALYs) is minimised [1, 2]. The values for alcohol consumption are derived from surveys. The WHO approach includes two dimensions, average level of consumption and patterns of drinking, the latter operationalised by prevalence of people with heavy drinking occasions [3], compared to lifetime abstention as the theoretical minimum [9]. For the overall population level, the aggregate indicator of adult alcohol *per capita* consumption (APC) is used, mainly based on recorded consumption—obtained from administrative data such as taxation records, or production, import, and export statistics of alcoholic beverages [10, 11]. Surveys are subsequently used to distribute the aggregate value by sex and age (for principles, see [12, 13]). As surveys underestimate real consumption in most cases (so-called “under-coverage” [14]), triangulation between APC and survey results will lead to an upshifting of survey consumption levels among drinkers, as information about abstainers (combined current and lifetime) is held constant [13]. Both approaches come to different conclusions regarding alcohol consumption and key outcomes (e.g., deaths estimated by [2] vs. [3] for the year 2019, the last year with estimates from both systems: 1.8 million vs. 2.6 million deaths, respectively). Thus, it seems necessary to examine the validity of the underlying methodologies used, and the overarching objective of this publication is to examine the methodology used by the WHO. As we cannot examine this globally, i.e., for all countries, it will be done in depth for just one region, the member states of the Association of South East Asian Nations (ASEAN [15]). This region was selected as it has high variability on economic development, level and patterns of alcohol consumption, and relevant cultural factors such as religion or alcohol policy [16]. An ongoing project on alcohol, alcohol control policy, economic development, and health in ASEAN countries provided the database and the expertise to examine the methodology used by the WHO to identify exposure [17]. This project includes representatives from each ASEAN Member State, which has enabled the collection of additional surveys from the 10 ASEAN countries. These surveys were also transmitted to WHO and were integrated into the next update of the WHO's monitoring system ([18]; for underlying methodology, see [19]). These efforts complemented the routine data collection conducted by country focal points [10]. To increase variability of countries even more, we added two comparison regions based on economic wealth, level and patterns of drinking, and cultural factors that affect alcohol consumption. For inclusion of the two additional regions, we relied on routine data collected by WHO only. What dimensions and variables will be crucial in this examination? Based on the literature, surveys introduce more potential biases than aggregate consumption figures [20, 21], especially, if aggregate consumption is mainly based on recorded consumption. While the problem of under-coverage may be mitigated by triangulation and upshifting, other problems arise from the stigmatisation of alcohol consumption in certain populations. This may lead survey respondents to claim abstinence to avoid admitting to consuming alcohol within populations or countries where there is a strong cultural norm of not consuming alcohol, such as in Muslim-majority countries (Brunei, Indonesia, Malaysia) [22, 23], where such an admission could lead to stigma and social embarrassment [24, 25]. If the prevalence of abstention is overestimated when using the WHO methodology, the level of alcohol consumption among drinkers will be overestimated, and alcohol-attributable harm may also be overestimated. Thus, checking the indicator of level of drinking per drinker can be considered one of the routine checks necessary to assess for potential bias. Based on the above considerations, this study aims to:

1. Based on WHO methodology to conduct CRAs for alcohol [3, 19], present key indicators of alcohol exposure for the 10 ASEAN countries for 2022 for the whole adult population and disaggregated by sex.
2. Critically examine the plausibility of these indicators, particularly the indicators measuring the level of drinking in people who consume alcohol, and discuss any reasons to question their validity.
3. Compare the results with those from two political and economic unions with differing wealth levels and drinking patterns: the European Union (EU) representing, with one exception, high-income countries (Table A1 in Appendix 1; gender-specific results in Table A2), and the East African Community (EAC), comprised of mainly low-income countries (Table A3 in Appendix 1; gender-specific results in Table A4).

## Methods

### Data sources

The year 2022 was the most recent year for which complete data were available. Economic data for each country were obtained from the World Bank (WB), including the gross domestic product *per capita* at purchasing power parity (GDP PPP *per capita*) in current international dollars [26] and the WB's income-level country classification [27]. All alcohol exposure-related data were obtained from the WHO [18] or calculated using WHO methodology: annual APC without tourist consumption, which is not relevant for the considerations here, as it did not exceed +/-0.2 litres for any of the ASEAN countries (Appendix 2); prevalence of abstention (i.e., people who have not consumed at least one standard drink of alcohol in the past 12 months); and prevalence of heavy episodic drinking (HED). To calculate APC per drinker in grams per day (g/day), an alcohol specific weight of 0.793 g/ml was used [28]. All indicator definitions follow WHO standards by excluding those under 15 years of age; HED is defined by consuming at least 60 g of pure alcohol on a single occasion in the past month [10]. This definition for HED was chosen by WHO to facilitate the estimation of alcohol-attributable burden for injuries and ischaemic diseases, which have been shown to be more related to HED than to the average level of alcohol consumption ([29, 30]; see exact WHO definitions of all indicators in [18]).

### Plausibility checks within countries

**Fig. 1:**
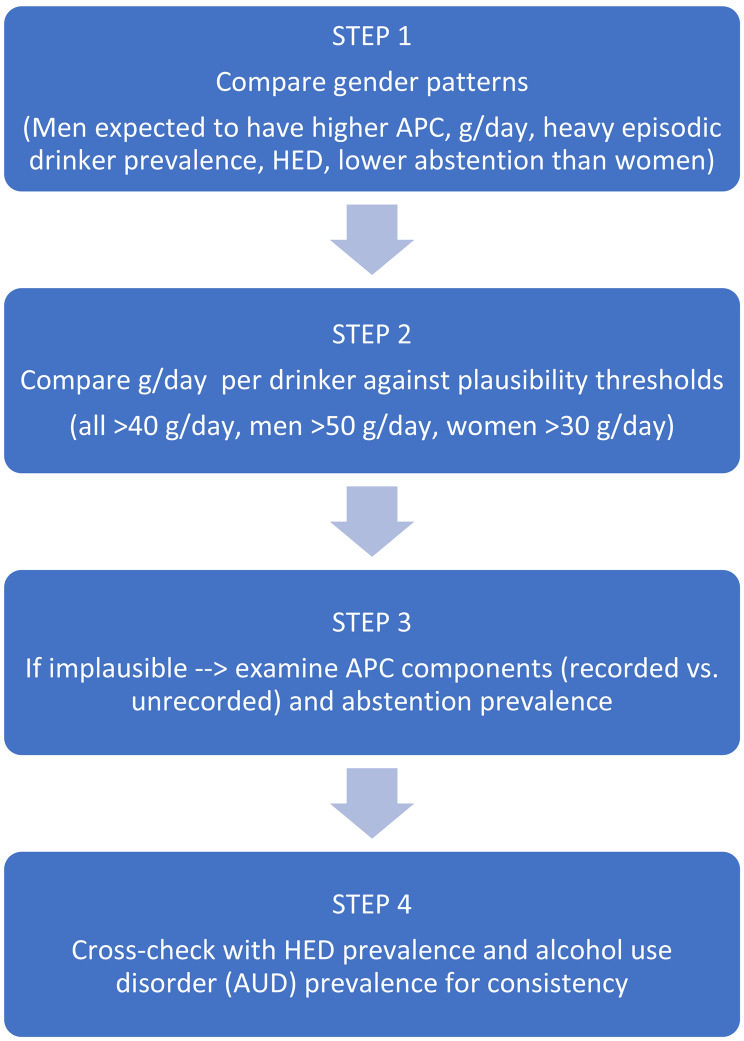
Plausibility check process for alcohol consumption estimates

A summary of this stepwise plausibility checking process is given in Fig. 1. First, we explored whether, when compared to women, men consistently show higher values for APC, average daily drinking level among drinkers, and prevalence of HED among drinkers, and lower values for abstainers [31, 32]. While the sex gap is narrowing in some populations [33], these patterns generally reflect traditional drinking behaviours, which prevail in ASEAN countries.

Second, as indicated above, the key measure for the plausibility checks is the average level of alcohol consumption per drinker. We routinely inspected all values above 40 grams of pure alcohol/day of this indicator for both genders combined, and above 30 and 50 grams of pure alcohol/day among women and men, respectively. The rationale is that, in ASEAN countries, daily or almost-daily alcohol consumption seems relatively rare in the general population; the drinking patterns tend to be intermittent, with occasional heavy drinking occasions, even in higher-consumption countries like the Lao People’s Democratic Republic (Lao PDR), Thailand, or Viet Nam [34–36]. In all three countries, on average people with alcohol consumption tend to drink between every second and every seventh day (e.g., Lao PDR 2020: 47 days/year [34]; Thailand 2012/13: 128 days/year [34]; Thailand 2021: 92 days/year [37]; Viet Nam 2014: 158 days/year [34]; Viet Nam 2014/15: 87 days/year [35]).

Therefore, average daily consumption levels exceeding these thresholds would imply very high amounts consumed per occasion—up to levels that are considered biologically improbable and have not been observed in surveys or any other research.

To help explain this unusually high level of average consumption per drinker at the national level, we examined the validity of the constituent parts of the indicator, i.e., APC, including both recorded and unrecorded consumption, the latter denoting any consumption of alcohol not registered in the country in which it is consumed [38], and prevalence of abstention. In addition, we evaluated the plausibility of these values by comparing them against the prevalence of HED among drinkers and the prevalence of alcohol use disorders (AUD).

### Simulations

To explore whether high values of drinking per drinking day among people who consume alcohol could be explained by small subgroups drinking very heavily, such as people with AUD [39–41], we conducted simulations. According to WHO [3] and GBD [42] estimates, the yearly prevalence of AUD in the three countries identified with values above the threshold was between 1 and 17% for the most recent years available (see Table A5 in Appendix 1 for details and confidence intervals). In addition, from recent surveys conducted in these three countries, the following values for 1-year prevalence of AUD were reported: Lao PDR (capital province): 7.5% for 2015 [43]; Thailand; 5.3% and 2.3% for 2013 and 2023, respectively [44, 45]. Based on these data, we simulated the level of use for those who consume alcohol—both with and without AUD—for three prevalences: 3%, 5%, and 10%, assuming either 100 g/day or 150 g/day for people with AUD. The average level of drinking for people with AUD was derived from the NESARC studies [39]; studies in the ASEAN countries indicate that these numbers may be too high for people with AUD in this region [45].

### Comparisons to other regions

For comparison, we present the same indicators for two regions with contrasting wealth levels and drinking patterns: the EU, a region composed mainly of high-income countries and very high levels of alcohol consumption [46] (see also Tables A1 and A2 in Appendix 1); and the EAC, a region composed mainly of low-income countries with low overall alcohol consumption but a high proportion of unrecorded consumption (for measurement of unrecorded and the link to wealth of countries, see [47]; see also Tables A3 and A4 in Appendix 1).

## Results

### Surveys identified

Appendix 3 lists the surveys identified and entered into the WHO monitoring framework (for methodology used, see [19]). A total of 96 key surveys were identified.

### Key indicators and plausibility checks

The resulting key indicators for the 10 ASEAN countries are presented in Tables 1 and **2**. Across all indicators for all countries, the predicted gaps between men and women were observed.

**Table 1:** Economic and alcohol exposure indicators for ASEAN member states, 2022

Country	GDP PPP per capita	World Bank income group	Adult per capita consumption (litres)	Percentage of unrecorded	Percentage of abstainers	Average consumption per day per drinker (g/day)	Prevalence of heavy episodic drinkers among drinkers
Brunei Darussalam	81,802	High	0.71	25%	60%*	3.85	34%
Indonesia	14,285	Upper-Middle	0.10	28%	88%	1.83	32%
Cambodia	6,919	Lower-Middle	6.37	12%	51%	28.32	40%
Lao People’s Democratic Republic	8,766	Lower-Middle	11.47	33%	41%	42.06	50%
Myanmar	5,732	Lower-Middle	1.87	9%	79%	18.89	44%
Malaysia	34,366	Upper-Middle	0.92	18%	79%	9.64	40%
Philippines	10,131	Lower-Middle	6.01	19%	52%	27.46	37%
Singapore	143,095	High	2.00	8%	29%	6.14	33%
Thailand	22,243	Upper-Middle	7.86	15%	64%	46.88	49%
Viet Nam	13,905	Lower-Middle	11.55	68%	39%	41.10	50%
**Population-weighted mean**	**15,582**	-	**4.08**	**28%**	**69%**	**29.01**	**43%**

Legend: GDP *per capita*, PPP, is given in current international $; WB: World Bank; APC: adult alcohol *per capita* consumption in litres pure alcohol; Average level per day for drinkers is measured in grams pure alcohol; HED: heavy episodic drinking

*Estimated by WHO methodology; the STEPS survey gives higher percentage

Grey-highlighted cells indicate values above the thresholds of 50 g/day and 30 g/day for men and women, respectively

All three Muslim-majority countries were well below the threshold of average alcohol consumption per drinker. However, three other ASEAN member states exceeded both the pre-defined thresholds for men and women combined and for men alone: Lao PDR, Thailand, and Viet Nam (Tables 1 and 2). We will first consider Thailand, which had the highest value for both genders combined (almost 47 g/day) and for men (almost 60 g/day, or six drinks per day, assuming a standard drink size of 10 g).

**Table 2:** Economic and alcohol exposure indicators for ASEAN member states by gender, 2022

Country	Gender	Adult per capita consumption (litres)	Percentage of abstainers	Average consumption per day for drinkers (g/day)	Prevalence of heavy episodic drinkers among drinkers
Brunei Darussalam	Men	1.18	49%*	5.02	35%
	Women	0.30	70%*	2.14	32%
Indonesia	Men	0.17	84%	2.22	34%
	Women	0.03	93%	0.85	24%
Cambodia	Men	9.95	41%	36.75	44%
	Women	2.49	62%	14.22	33%
Lao People’s Democratic Republic	Men	18.24	31%	57.73	56%
	Women	4.69	50%	20.45	42%
Myanmar	Men	3.14	70%	23.09	48%
	Women	0.57	87%	9.28	35%
Malaysia	Men	1.61	71%	12.07	44%
	Women	0.30	87%	4.90	33%
Philippines	Men	9.53	42%	35.92	41%
	Women	2.41	63%	14.06	31%
Singapore	Men	3.09	22%	8.63	45%
	Women	0.99	36%	3.33	18%
Thailand	Men	12.87	53%	59.18	54%
	Women	2.50	75%	21.85	39%
Viet Nam	Men	18.05	30%	55.78	56%
	Women	4.56	49%	19.41	41%
**Population-weighted mean**	**Men**	**6.61**	**61%**	**37.28**	**47%**
**Population-weighted mean**	**Women**	**1.50**	**78%**	**14.53**	**35%**

Legend: APC: adult alcohol *per capita* consumption in litres pure alcohol; Average level per day for drinkers is measured in grams pure alcohol; HED: heavy episodic drinking

*Estimated by WHO methodology; the STEPS survey gives higher percentage

Grey-highlighted cells indicate values above the threshold of 40 g/day

This average level of alcohol consumption among drinkers seems biased in Thailand. If the indicator was unbiased, it would imply that Thai men consuming alcohol have a heavy drinking occasion, as defined by the WHO, almost every day.

In reality, Thai men consuming alcohol, based on a large general population survey conducted in 2021 [48], drank on average twice per week (104 days/year [34, 37]). This would mean that their average drinking per occasion (i.e., drinking day) would represent a very high level, at around 21 drinks for every heavy drinking occasion. Such average high levels of drinking per occasion in Thai men, however, have not been reported in any population survey from Thailand.

For both genders combined, the numbers would be as follows: average level of drinking per person drinking: 47 g/day (Table 1); average consumption per drinking day based on 92 days/year (or about every fourth day) [37]: about 19 drinks on average per Thai drinker.

### Simulations

Table 3 gives an overview of the results of the simulation modelling. Overall, the simulations indicate that it would take the more extreme scenarios—i.e., 10% of the general population with AUD and on average 150 g/day combined with at least 183 drinking occasions—to produce numbers that may be considered plausible (see green-highlighted cells in Table 3). However, even under these somewhat unrealistic conditions, the numbers could still be considered to be somewhat on the high side. In other words, we would consider the values of the average level of drinking indicator to still be biased for all three countries.

**Table 3:** A comparison between the available data and simulated estimates on drinking per drinking day, 2022

	Data from WHO					Simulated estimates based on the available data			
	Total alcohol per capita	% unrecorded	% abstainer/% drinker last 12 months	Average consumption per day for drinkers in g/day	Prevalence of AUD in general population	Drinking for drinkers with AUD in g/day	Drinking for drinkers without AUD in g/day	Drinking per drinking day in grams − 183 days	Drinking per drinking day in grams − 122 days
Lao PDR	11.47	33	41/59	42.1	3%	100	39	78	117
					3%	150	36	73	109
					5%	100	37	73	110
					5%	150	32	64	96
					10%	100	30	61	91
					10%	150	20	40	60
Thailand	7.86	15	64/36	46.9	3%	100	42	84	126
					3%	150	38	75	113
					5%	100	38	77	115
					5%	150	30	61	91
					10%	100	26	53	79
					10%	150	7	14	22
Viet Nam	11.55	68	39/61	41.1	3%	100	38	76	114
					3%	150	35	71	106
					5%	100	36	72	108
					5%	150	31	63	94
					10%	100	30	59	89
					10%	150	20	39	59

### Examining underlying indicators

Given this likely bias, we examined the indicator’s constituents. Thailand’s APC is above the global average, but markedly lower than the APC in Lao PDR or Viet Nam. Most of the APC in Thailand is recorded consumption, which is arguably the most reliable part of APC as the data are derived from the taxation system [11, 20]. Thus, the prevalence of abstainers is most likely overestimated in Thai surveys, and is presumably the main source of bias in the indicator for average level of consumption for drinkers. In 2022, the WHO estimated 64% abstainers, 53% among men, and 75% among women (Table 2). However, all major surveys in the past years from Thailand show even higher abstention rates, most often more than 70% [49], indicating that via the WHO methodology, the survey abstainer values were already been corrected downwards by other predictors of the estimation used [19].

For the Lao PDR and Viet Nam, the average alcohol consumption per drinker is similar to that in Thailand (Tables 1 and 2), and the same overall considerations apply. The simulation study also shows similar results, suggesting that the indicators for both Lao PDR and Viet Nam are likely biased upwards, albeit less so than for Thailand. The differences between the three countries are mainly explained by the overall higher APC in the Lao PDR and Viet Nam.

Regarding the constituents, in both the Lao PDR and even more so in Viet Nam, the proportion of unrecorded alcohol constitutes a large share of total consumption, 33% in the Lao PDR and 68% in Viet Nam. These values, mainly derived from the regularly conducted WHO STEPS Surveys [50] (Lao PDR: 2008, 2013, 2020; Viet Nam: 2009, 2015, 2021), are consistent with other survey data (Appendix 3). The STEPS Survey globally is the standard tool for assessing unrecorded consumption [51]. Overall, both the unrecorded estimates and the abstainer prevalence are based on survey data, and both survey questions could be considered to be asking about potentially stigmatised behaviour, so it is harder to decide where we suspect the source of the bias for the Lao PDR and Viet Nam to emanate from, compared to Thailand.

### Comparisons to other regions

**Fig. 2:**
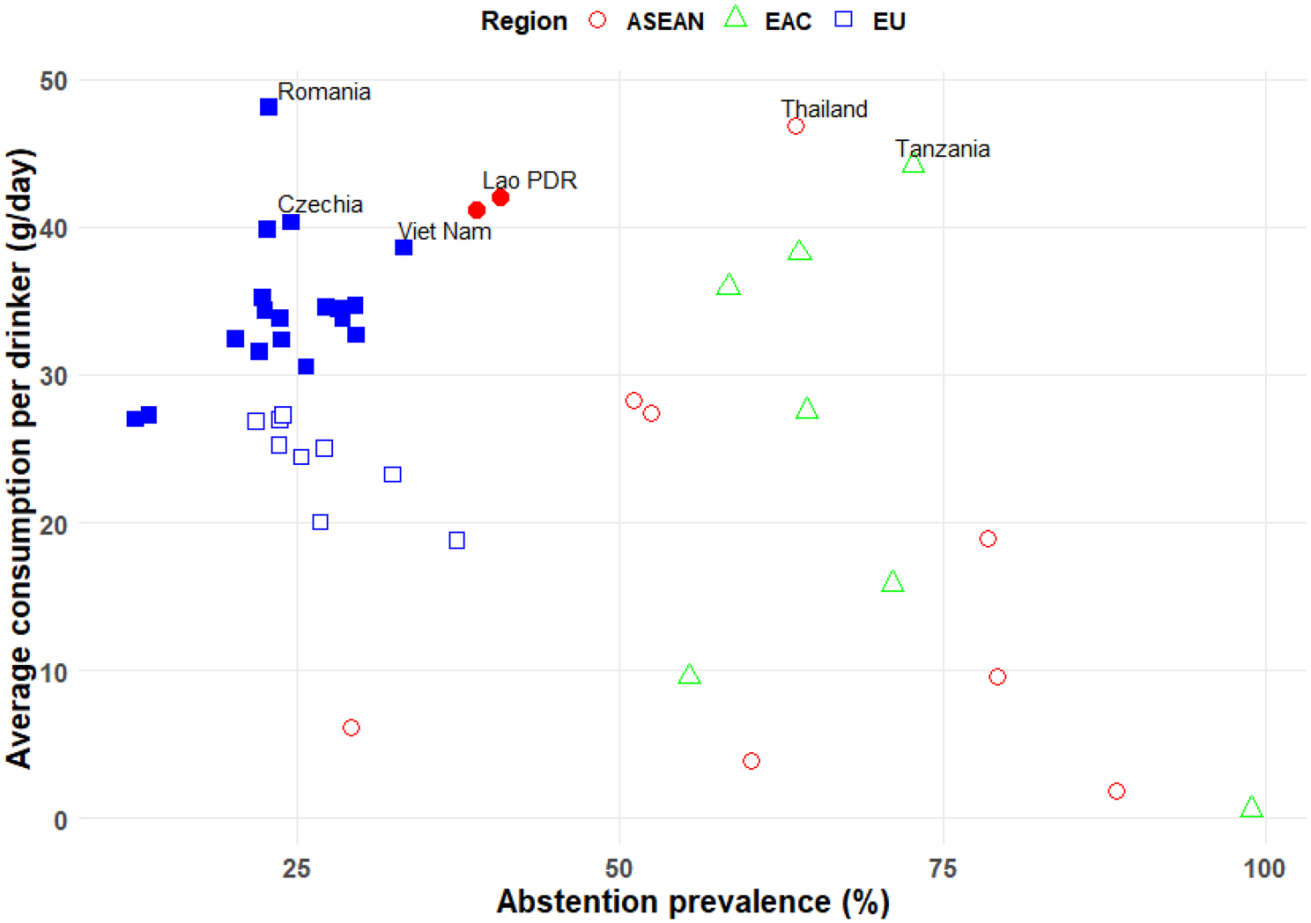
Scatterplot of prevalence of 12-month abstention with average level of alcohol consumption among drinkers

Three out of 10 ASEAN member states had values for the indicator of average level of alcohol consumption among drinkers that looked potentially biased. What is the situation in the two comparison regions? In the EU, which has 27 member states, there are two countries which surpass the thresholds for both genders: Romania and Czechia (Table A1 in Appendix 1). Both countries fall within the 10 countries globally that have the highest APC: Romania with the highest APC globally, and Czechia ranked as 6^th^. Both countries also have APC values which are at least 20% higher than the highest APC of the ASEAN member states (Romania 48% higher; Czechia 21% higher). Thus, being above the threshold for the level of alcohol consumption among drinkers of 40 g/day in a country like Romania, where the average drinking level among the general population is already 37 g/day (equivalent to 17 l APC), is not implausible (for a graphical representation, see Fig. 2).

Overall, as indicated above, the EU is the region with the highest level of alcohol consumption globally. Given this very high level of drinking, the values for the indicator seem less problematic, including for Czechia, which barely exceeded the threshold. With respect to gender-specific values, the following countries surpassed the threshold for men (Table A2 in Appendix 1 ): Bulgaria, Czechia, Latvia, and Romania, with the following ranks regarding APC (12^th^, 6^th^, 5^th^ and 1^st^, respectively). They are also all in the Eastern part of the EU, characterised by larger differences in drinking levels between women and men.

In sum, while the values for the indicators for the level of alcohol consumption among drinkers pass the threshold set a priori, we cannot exclude the possibility that these values are actually unbiased, given the high overall levels of drinking within the EU.

As for the EAC, there is only one country with levels above the thresholds for both genders and for men (but not for women): Tanzania. Out of all the countries with thresholds set for the indicator average level of drinking among drinkers, the APC of Tanzania is the lowest, at 5.5 l pure alcohol. Approximately 73% of Tanzanian adults are abstainers, in part for religious reasons, as more than one third of the inhabitants of Tanzania are Muslim [52]. As indicated above, this may be one of the reasons for the low value. Another potential reason is the relatively high proportion of unrecorded consumption, which may be overestimated (48%; see Table A3 in Appendix 1 ). The last STEPS survey showed lower proportions of unrecorded consumption and higher rates of abstention [53]. Finally, no part of APC is based on administrative data such as taxation, but rather is estimated from food balance sheets produced by the Food and Agriculture Organization of the United Nations (FAO), which is considered to be potentially much more biased that the former sources [10].

The association between prevalence of abstention and level of alcohol consumption among drinkers in different regions is further illustrated in Fig. 2, which shows a scatterplot of abstention prevalence versus g/day per drinker for ASEAN, EU, and EAC countries, highlighting how a reported low prevalence of current drinking can inflate the per-drinker consumption estimate, especially in countries with an overall lower APC.

## Discussion

The WHO current methodology for estimating alcohol exposure in CRAs was tested for plausibility across all ASEAN member states using the latest available indicators. It was found that the indicator on average volume of alcohol consumption among people who consume alcohol showed implausibly high values for three countries. Further simulations based on assumptions regarding the prevalence of people with heavy or very heavy drinking levels further corroborated a likely bias in these values. An examination of its constituents revealed that the problem for Thailand is likely based on survey answers to the question on alcohol abstention, in which a high number of respondents claimed no consumption of alcohol during the past 12 months, but had likely consumed some alcohol during this time period. For the Lao PDR and Viet Nam, the same problem seems to exist, but we cannot exclude the possibility that survey answers to the question regarding unrecorded alcohol may also have contributed. Investigations of two other regions also showed some implausible values.

Before we discuss these findings further, we need to discuss potential limitations of our approach. First, the results of every empirical investigation are only as valid as the underlying empirical data. In our case, the data on alcohol exposure are based on different sources: administrative data, modelling, and surveys. While administrative data is considered to be the most reliable, alcohol sold in a jurisdiction is only an indirect indicator of exposure. Not all alcohol is consumed; there is some waste due to broken or leaky containers, containers not fully emptied, spillage, etc. This is why the Technical Advisory Group for Alcohol and Drug Epidemiology of WHO [54] recommends that only 80% of the total APC should be included in the WHO's burden estimates. It may be advisable to base the APC in the indicator on the average level of alcohol consumed among drinkers on this lower value. All modelling is based on assumptions, and the assumptions to derive APC from food balance sheets are manyfold, leading to relatively high variability of annual estimates by the FAO [55]. However, for many low- and lower-middle income countries in Africa, which are not considered big markets for industry, FAO estimates are the only source for APC data [3]. Finally, while great care was taken to identify all relevant surveys for the countries under investigation, there may still be some systematic errors stemming from surveys themselves, as they are based on self-report with incomplete survey frames [21, 56]. The problem is further complicated as there is a concentration of drinking, where a minority of the drinking population consumes the majority of the alcohol [57], and this minority is often not part of the sampling frame [58]. So, clearly, there is work to be done to explore and correct for the most important biases resulting from surveys.

In sum, the WHO methodology offers advantages for estimating alcohol exposure for CRAs, as it includes two relevant dimensions that have been shown to impact burden [30], and as it is anchored in the overall most reliable and valid indicator: APC [20]. However, care must be taken in the triangulation of this indicator with survey results, especially for the indicator of abstention. The reliability of this indicator seems to vary based on culture and stigmatisation of alcohol consumption. If abstention is markedly overestimated, this will in turn cause an overestimate of the number of people with very high alcohol consumption, potentially resulting in inflated burden estimates [59]. Finally, we used only one threshold value to detect potential problems in the indicator of average level of drinking among drinkers. This level may be too high for Muslim-majority countries, where overall drinking levels have been shown to be low in ASEAN countries. If only a few drinking occasions per year are reported, lower thresholds than 40 g/day should be used. However, the estimated levels of drinking for all three ASEAN Muslim countries were all below 10 g/day, so this consideration is not relevant for this region.

What are the implications of our results for GBD's work on alcohol [1, 60], which is entirely based on survey-derived alcohol estimates? First, the mechanism described, which may lead to claim normative drinking status—i.e., being an abstainer in a culture where abstention is the norm, or claiming to be a drinker in cultures where drinking is the norm—applies to the GBD methodology as well. Second, unique to GBD, their estimate will reflect the survey underestimation of real drinking levels described above. Real drinking levels denote the levels mainly defined based on sold and taxed alcohol [10, 11, 15]. In sum, the GBD methodology will suffer more biases than the WHO methodology and will result in an underestimate of consumption and attributable harm. It has been argued that the risk estimates for disease and mortality outcomes are also based on self-reports and thus underestimated alcohol consumption. However, clinical and other cohorts and the instruments used in such studies have been shown to underestimate the level of consumption less than general population surveys on alcohol ([61]; for a review [62]), and the underestimation of population level is not only caused by the sum of individual underestimation, but also by insufficient sampling frames, leaving out the heavy and very heavy consumers [21, 58] who are responsible for the majority of all alcohol consumed [57]. Thus, the current WHO methodology based on triangulation seems to be the better methodology [62].

What can be done to reduce the potential bias detected in this study? Take Thailand again as an example. The large surveys in this country all show high rates of abstention, often over 70% [49], which seems to be implausible, given the country’s APC. Future research should look into the methodology used in these surveys, and perform cognitive interviews to improve the questions on abstention [63]. It could be that despite the fact that the Thai form of Buddhism does not seem to hinder heavy drinking, there may still be some implicit norms acting and impacting on respondents’ answers. For example, when modern community campaigns reinforced traditional values during the Buddhist Lent period, consumption decreased during these months [64]. Alternatively, respondents may see drinking as something fairly common and acceptable and thus discount the occasional drinking of alcohol, still considering themselves to be abstainers. Only further research, such as performing cognitive interviews, will be able to answer these questions and improve future surveys.

In conclusion, methodological research, which continuously tests the assumptions of currently used monitoring systems such as the WHO monitoring system, is necessary if we want to base policy decisions on the best evidence available.

## Data Availability

All sources of data are publicly available, and the sources are specified in the text. Moreover, the data for the plausibility underlying the plausibility checks are given in the Tables of the manuscript or in the Appendices.

## Appendix 1 to: Defining Exposure for Estimating the Global Burden of Alcohol Consumption: Plausibility Testing of WHO Methodology Using ASEAN Countries as a Case Study

**Table A1:** Economic and alcohol exposure indicators for European Union countries 2022

Country	GDP PPP per capita	World Bank income group	Adult per capita consumption (litres)	Percent unrecorded	Percent abstainers	Average consumption per day for drinkers (g/day)	Prevalence of heavy episodic drinkers among drinkers
Austria	70,735	High	11.91	6%	20%	32.44	32%
Belgium	68,158	High	8.41	7%	25%	24.46	31%
Bulgaria	35,814	Upper-Middle	11.86	7%	33%	38.62	23%
Cyprus	55,942	High	5.43	14%	37%	18.84	23%
Czechia	51,710	High	14.02	4%	25%	40.36	42%
Germany	67,590	High	11.32	6%	22%	31.57	30%
Denmark	77,400	High	9.67	6%	22%	26.89	35%
Spain	50,468	High	12.27	6%	23%	34.40	30%
Estonia	47,802	High	10.46	8%	26%	30.58	42%
Finland	61,347	High	9.49	7%	24%	27.01	38%
France	56,181	High	11.36	6%	24%	32.41	28%
Greece	38,969	High	7.24	12%	32%	23.27	25%
Croatia	41,956	High	11.25	8%	30%	34.68	34%
Hungary	44,012	High	11.38	7%	28%	34.46	40%
Ireland	136,104	High	10.87	4%	14%	27.33	47%
Italy	56,247	High	8.40	5%	27%	25.04	30%
Lithuania	50,498	High	11.88	5%	24%	33.82	44%
Luxembourg	143,382	High	10.88	3%	13%	27.04	43%
Latvia	39,961	High	14.18	19%	23%	39.84	41%
Malta	60,485	High	6.76	10%	27%	20.07	42%
Netherlands	77,152	High	8.88	7%	24%	25.27	33%
Poland	46,077	High	11.59	11%	27%	34.62	41%
Portugal	44,557	High	12.59	6%	22%	35.24	39%
Romania	42,215	High	17.08	33%	23%	48.11	40%
Slovakia	41,096	High	11.11	8%	29%	33.79	40%
Slovenia	51,079	High	10.61	8%	30%	32.72	31%
Sweden	66,376	High	9.57	20%	24%	27.34	31%
**Population-weighted mean**	**57,512**	−	**11.04**	**8%**	**24%**	**31.75**	**33%**

Legend: GDP *per capita*, PPP, is given in current international $; WB: World Bank; APC: adult alcohol *per capita* consumption in litres pure alcohol; Average level per day for drinkers is measured in grams pure alcohol; HED: heavy episodic drinking

Grey-highlighted cells indicate values above the threshold of 40 g/day

**Table A2:** Economic and alcohol exposure indicators for European Union countries by gender 2022

Country	Gender	Adult per capita consumption (litres)	Percent abstainers	Average consumption per day for drinkers (g/day)	Prevalence of heavy episodic drinkers among drinkers
Austria	Men	18.22	14%	45.96	41%
	Women	5.30	27%	15.75	22%
Belgium	Men	12.88	18%	33.98	40%
	Women	3.74	33%	12.18	20%
Bulgaria	Men	18.31	25%	52.89	28%
	Women	4.85	43%	18.33	16%
Cyprus	Men	8.55	27%	25.37	28%
	Women	2.33	48%	9.72	16%
Czechia	Men	21.62	18%	57.22	50%
	Women	6.08	31%	19.29	32%
Germany	Men	17.41	15%	44.56	39%
	Women	5.00	29%	15.36	20%
Denmark	Men	14.84	15%	37.97	44%
	Women	4.38	29%	13.37	23%
Spain	Men	18.88	15%	48.48	38%
	Women	5.31	30%	16.49	21%
Estonia	Men	15.49	20%	42.22	51%
	Women	4.71	32%	15.03	31%
Finland	Men	14.54	16%	37.80	47%
	Women	4.26	31%	13.45	26%
France	Men	17.28	17%	45.04	35%
	Women	4.92	32%	15.65	19%
Greece	Men	11.11	23%	31.32	31%
	Women	3.04	43%	11.52	16%
Croatia	Men	17.16	22%	47.75	41%
	Women	4.76	38%	16.64	25%
Hungary	Men	17.23	21%	47.45	48%
	Women	4.88	36%	16.63	30%
Ireland	Men	16.42	10%	39.46	58%
	Women	5.12	18%	13.53	34%
Italy	Men	12.87	19%	34.47	38%
	Women	3.64	36%	12.34	19%
Lithuania	Men	17.52	19%	46.80	52%
	Women	5.34	30%	16.46	33%
Luxembourg	Men	16.58	9%	39.51	54%
	Women	5.24	16%	13.57	31%
Latvia	Men	20.78	18%	54.93	48%
	Women	6.20	29%	18.84	31%
Malta	Men	10.66	18%	28.37	54%
	Women	3.15	35%	10.48	29%
Netherlands	Men	13.65	16%	35.45	42%
	Women	3.99	31%	12.58	21%
Poland	Men	17.67	20%	48.06	49%
	Women	4.99	35%	16.67	30%
Portugal	Men	19.06	15%	48.93	47%
	Women	5.33	30%	16.59	28%
Romania	Men	26.14	17%	68.15	47%
	Women	7.24	30%	22.34	31%
Slovakia	Men	17.05	21%	46.91	48%
	Women	4.77	37%	16.35	29%
Slovenia	Men	16.59	22%	46.01	38%
	Women	4.64	37%	16.09	22%
Sweden	Men	14.86	16%	38.63	40%
	Women	4.31	31%	13.65	21%
**Population-weighted mean**	**Men**	**16.91**	**17%**	**44.38**	**40%**
**Population-weighted mean**	**Women**	**4.80**	**32%**	**15.38**	**22%**

Legend: APC: adult alcohol *per capita* consumption in litres pure alcohol; Average level per day for drinkers is measured in grams pure alcohol; HED: heavy episodic drinking

Grey-highlighted cells indicate values above the thresholds of 50 g/day and 30 g/day for males and females, respectively

**Table A3:** Economic and alcohol exposure indicators for East African community states 2022

Country	GDP PPP per capita	World Bank income group	Adult per capita consumption (litres)	Percent unrecorded	Percent abstainers	Average consumption per day for drinkers (g/day)	Prevalence of heavy episodic drinkers among drinkers
Burundi	889	Low	4.50	29%	65%	27.54	57%
Democratic Republic of the Congo	1,484	Low	1.97	50%	55%	9.58	82%
Kenya	5,883	Lower-Middle	2.11	21%	71%	15.87	52%
Rwanda	3,099	Low	6.87	64%	58%	35.91	60%
Somalia	1,487	Low	0.00	0%	99%	0.59	42%
Tanzania	3,800	Lower-Middle	5.55	48%	73%	44.20	48%
Uganda	2,919	Low	6.35	41%	64%	38.23	50%
**Population-weighted mean**	**2,985.42**	-	**3.62**	**0.40**	**0.66**	**23.23**	**0.64**

Legend: GDP *per capita*, PPP, is given in current international $; WB: World Bank; APC: adult alcohol *per capita* consumption in litres pure alcohol; Average level per day for drinkers is measured in grams pure alcohol; HED: heavy episodic drinking

Grey-highlighted cells indicate values above the threshold of 40 g/day

**Table A4:** Economic and alcohol exposure indicators for East African community states by gender 2022

Country	Gender	Adult per capita consumption (litres)	Percent abstainers	Average consumption per day for drinkers (g/day)	Prevalence of heavy episodic drinkers among drinkers
Burundi	Men	7.22	55%	35.03	61%
	Women	1.68	74%	14.12	50%
Democratic Republic of the Congo	Men	3.07	45%	12.20	83%
	Women	0.83	66%	5.26	79%
Kenya	Men	3.43	62%	19.83	56%
	Women	0.76	80%	8.22	45%
Rwanda	Men	10.81	49%	46.03	64%
	Women	2.56	69%	17.83	52%
Somalia	Men	0.01	98%	0.72	45%
	Women	0.00	99%	0.25	33%
Tanzania	Men	9.07	64%	55.35	52%
	Women	1.86	81%	21.79	39%
Uganda	Men	10.17	55%	48.88	54%
	Women	2.37	73%	19.37	43%
**Population-weighted mean**	**Men**	**5.84**	**0.57**	**29.74**	**0.67**
**Population-weighted mean**	**Women**	**1.33**	**0.75**	**11.62**	**0.59**

Legend: APC: adult alcohol *per capita* consumption in litres pure alcohol; Average level per day for drinkers is measured in grams pure alcohol; HED: heavy episodic drinking

Grey-highlighted cells indicate values above the thresholds of 50 g/day and 30 g/day for males and females, respectively

**Table A5:** Estimated prevalence of alcohol use disorders from the World Health Organization and the global burden of Disease study

Study/country	Year	Gender	Alcohol use disorders			Alcohol dependence		
			Point estimate	lower 95% CI	upper 95% CI	Point estimate	lower 95% CI	upper 95% CI
**World Health Organization**								
Lao People’s Democratic Republic	2020	Women	12.05%	6.34%	17.76%	6.49%	3.00%	9.98%
		Men	22.75%	14.57%	30.92%	12.95%	7.29%	18.61%
		Both	17.42%	10.47%	24.36%	9.73%	5.15%	14.31%
Thailand	2020	Women	5.08%	2.84%	7.32%	2.54%	1.25%	3.82%
		Men	12.78%	8.59%	16.98%	6.69%	3.92%	9.46%
		Both	8.78%	5.60%	11.96%	4.53%	2.53%	6.53%
Viet Nam	2020	Women	7.58%	3.93%	11.23%	3.90%	1.80%	5.99%
		Men	17.50%	11.36%	23.64%	9.53%	5.49%	13.56%
		Both	12.39%	7.53%	17.24%	6.62%	3.59%	9.66%
**Global Burden of Disease study**								
Lao People’s Democratic Republic	2021	Women				0.34%	0.27%	0.42%
		Men				1.55%	1.23%	1.93%
		Both				0.94%	0.76%	1.16%
Thailand	2021	Women				0.34%	0.27%	0.42%
		Men				2.30%	1.88%	2.77%
		Both				1.28%	1.06%	1.52%
Viet Nam	2021	Women				0.35%	0.27%	0.44%
		Men				2.31%	1.86%	2.82%
		Both				1.31%	1.07%	1.58%

Note: Estimates of WHO are for people 15+ years; GBD estimates are for 20+ years. The GBD numbers are for AUD, but as they exclude less severe AUD without disability weight, de facto the prevalence applies more to alcohol dependence as defined in ICD 10 or 11

## Appendix 2: Estimated adult tourist alcohol *per capita* consumption of ASEAN member states in 2022 in litres pure alcohol for population 15 and older

**Table:**

Year	Brunei Darussalam	Indonesia	Cambodia	Lao PDR	Myanmar	Malaysia	Philippines	Singapore	Thailand	Viet Nam
**2022**	0	0	−0.0110	0.0049	0.0176	0	0.0137	−0.1606	−0.0136	0.0132

Negative numbers denote, that incoming tourists consume more alcohol than outgoing tourists; positive numbers indicate the contrary; and 0 means that the net difference in consumption between incoming and outgoing tourists was smaller than │± 0.00005│

## Appendix 3: Sources of alcohol patterns of consumption data by the World Health Organization (WHO) Region and Association of Southeast Asian Nations (ASEAN) member states

**Table:**

Country	Sources
**Region: WHO South-East Asia Region (SEAR)**	
**Indonesia**	Ministry of Home Affairs, National Development Planning Agency, Statistics Indonesia & United Nations Children’s Fund 2013. Indonesia - West Papua Multiple Indicator Cluster Survey 2011. New York, USA: United Nations Children’s Fund.
	Statistics Indonesia (Badan Pusat Statistik—BPS), National Population and Family Planning Board (BKKBN), Kementerian Kesehatan (Kemenkes—MOH), ICF International (2013). Indonesia Demographic and Health Survey 2012. Jakarta, Indonesia: BPS, BKKBN, Kemenkes, and ICF International.
	Indonesia Agency of Health Research and Development of Ministry of Health of Republic of Indonesia. (2018). 2018 National Basic Health Research (Riskesdas) Report. Jakarta, Indonesia: Ministry of Health of Republic of Indonesia.
	Indonesia Agency of Health Development Policy of Ministry of Health of Indonesia. (2023). Indonesia Health Survey 2023 in Numbers. Jakarta, Indonesia: Ministry of Health of Indonesia.
**Myanmar**	World Health Organization (2004, urban). STEPwise approach to surveillance (STEPS) Survey.
	World Health Organization (2004, rural). STEPwise approach to surveillance (STEPS) Survey.
	World Health Organization (2009). STEPwise approach to surveillance (STEPS) Survey.
	World Health Organization (2014b). STEPwise approach to surveillance (STEPS) Survey.
	Bjertness MB, Htet AS, Meyer HE, Htike MM, Zaw KK, Oo WM, et al. (2016). Prevalence and determinants of hypertension in Myanmar - a nationwide cross-sectional study, BMC Public Health.16:590.
**Thailand**	National Statistical Office, Ministry of Information and Communication Technology (2009) Survey on Health and Welfare. https://www.nso.go.th/nsoweb/nso/statistics_and_indicators?impt_branch=305. Accessed June 2025
	National Statistical Office (2011). Cigarette Smoking and Drinking Behaviour Survey: Alcohol Drinking Habit.
	Greenfield TK, Bloomfield K, Wilsnack SC (2013d). GENAHTO Project (Gender and Alcohol’s Harm to Others). (http://genahto.org/. Accessed: 07/02/2020).
	National Statistical Office, Ministry of Information and Communication Technology (2013) Survey on Health and Welfare. https://www.nso.go.th/nsoweb/nso/statistics_and_indicators?impt_branch=305. Accessed June 2025
	National Statistical Office (2014). Cigarette Smoking and Drinking Behaviour Survey: Statistical Tables.
	Bureau of Non-communicable Diseases, Department of Disease Control, Ministry of Public Health (2015). Core Table: Behavioural Risk Factor Surveillance System 2015 (BRFSS 2015). (http://www.thaincd.com/2016/media-detail.php?id=10520%26tid=%26gid=1-015-005. Accessed: 10/22/2019).
	Sirirassamee T, Sirirassamee B (2015). Health risk behavior among Thai youth: national survey 2013, Asia-Pacific Journal of Public Health.27 [1]: 76–84. https://dx.doi.org/10.1177/1010539514548759.
	World Health Organization, Centers for Disease Control and Prevention (2015). Global School-based Student Health Survey – Thailand 2015 Fact Sheet.
	Intarut N, Pukdeesamai P (2017). Socioeconomic Inequality in Concurrent Tobacco and Alcohol Consumption. Asian Pacific Journal for Cancer Prevention.18 [7]: 1913–1917.
	Tanaree A, Assanangkornchai S, Kittirattanapaiboon P (2017). Pattern and risk of developing alcohol use disorders, illegal substance use and psychiatric disorders after early onset of alcohol use: Results of the Thai National Mental Health Survey 2013, Drug and Alcohol Dependence.170(ebs, 7,513,587):102–11. https://dx.doi.org/10.1016/j.drugalcdep.2016.11.001.
	National Statistical Office (2018). The smoking and drinking behaviour survey 2017.
	Saengow U (2019). Drinking abstinence during a 3-month abstinence campaign in Thailand: weighted analysis of a national representative survey, BMC Public Health.19 [1]: 1688. https://dx.doi.org/10.1186/s12889-019-8051-z.
	National Statistical Office, Ministry of Digital Economy and Society (2021). The 2021 health behaviour of population survey (https://www.nso.go.th/nsoweb/storage/survey_detail/2023/20230505110449_84709.pdf. Accessed: 03/06/2024).
	World Health Organization, Centers for Disease Control and Prevention (2023). Global School-based Student Health Survey – Thailand 2021 Fact Sheet. (https://extranet.who.int/ncdsmicrodata/index.php/catalog/946. Accessed: 01/28/2024).
**Region: WHO Western Pacific Region (WPR)**	
**Brunei Darussalam**	World Health Organization (2016). STEPwise approach to surveillance (STEPS) Survey.
	World Health Organization, Centers for Disease Prevention and Control (2014). Global School-based Student Health Survey – Brunei Darussalam 2014 Fact Sheet.
	World Health Organization, Centers for Disease Control and Prevention (2023). Global School-based Student Health Survey – Brunei Darussalam 2019 Fact Sheet. (https://extranet.who.int/ncdsmicrodata/index.php/catalog/940. Accessed: 04/02/2024).
**Cambodia**	National Institute of Statistics (NIS) [Cambodia], Ministry of Health (MoH) [Cambodia], ICF (2023). Cambodia Demographic and Health Survey 2021–22 Final Report. (https://dhsprogram.com/publications/publication-FR377-DHS-Final-Reports.cfm. Accessed: 01/26/2024).
	Schunert T, Khann S, Kao S, Pot C, Saupe LB, Sek S, et al. (2012). Cambodian Mental Health Survey 2012. (http://tpocambodia.org/wp-content/uploads/2015/09/Cambodian-Mental-Health-Survey-2012-RUPP.pdf. Accessed: 06/16/17).
	World Health Organization (2010). STEPwise approach to surveillance (STEPS) Survey.
	World Health Organization, Centers for Disease Control and Prevention (2014). Global School-based Student Health Survey – Cambodia 2013 Fact Sheet.
**Lao People’s Democratic Republic**	World Health Organization (2008). STEPwise approach to surveillance (STEPS) Survey.
	Greenfield TK, Bloomfield K, Wilsnack SC (2013). GENAHTO Project (Gender and Alcohol’s Harm to Others). (http://genahto.org/. Accessed: 07/02/2020).
	World Health Organization (2013). STEPwise approach to surveillance (STEPS) Survey.
	Lao Statistics Bureau (2018). Lao Social Indicator Survey II 2017, Survey Findings Report. Vientiane, Lao PDR: Lao Statistics Bureau, UNICEF.
	World Health Organization (2020). STEPwise approach to surveillance (STEPS) Survey.
	Lao PDR, National Health Survey 2020
	Lao Statistics Bureau (2024). Lao Social Indicator Survey 2023 – Key Indicators Report. (https://www.unicef.org/laos/media/9981/file/LSIS%20III-MICS%202023%20Key%20Indicators%20Report%20EN%2006%2002%202024%20Final.pdf. Accessed: 01/27/2024).
**Malaysia**	Institute for Public Health (2011). National Health and Morbidity Survey 2011 (NHMS 2011). Vol. II: Non-Communicable Diseases. Kuala Lumpur, Malaysia: Institute for Public Health.
	Centers for Disease Control and Prevention, Ministry of Health, World Health Organization (2012). Malaysia Global School-based Student Health Survey 2012. Geneva, Switzerland: World Health Organization.
	Institute for Public Health (2015). National Health and Morbidity Survey 2015 (NHMS 2015). Vol. II: Non-Communicable Diseases, Risk Factors & Other Health Problems. Malaysia, Kuala Lumpur: Institute for Public Health.
	Institute for Public Health, National Institutes of Health, Ministry of Health Malaysia (2020). National Health and Morbidity Survey (NHMS) 2019. Vol. I: NCDs – Non-Communicable Diseases: Risk Factors and other Health Problems. Kuala Lumpur, Malaysia: Institute for Public Health, National Institutes of Health, Ministry of Health.
	Institute for Public Health, National Institutes of Health, Ministry of Health Malaysia, Kuala Lumpur (2022). National Health and Morbidity Survey 2022- Adolescent Health Survey 2022. (https://iku.gov.my/images/nhms-2022/Report_Malaysia_nhms_ahs_2022.pdf. Accessed: 03/26/2024).
	Institute for Public Health, National Institutes of Health, Ministry of Health Malaysia (2023). National Health and Morbidity Survey (NHMS) 2023. Vol. I: NCDs – Non-Communicable Diseases: Risk Factors and other Health Problems. Kuala Lumpur, Malaysia: Institute for Public Health, National Institutes of Health, Ministry of Health.
**Philippines**	Philippine Statistics Authority (PSA) and ICF. (2003). 2003 Philippine National Demographic and Health Survey (NDHS).
	Philippines Global School-based Student Health Survey, 2003
	Philippines Global School-based Student Health Survey, 2007
	Philippine Statistics Authority (PSA) and ICF. (2008). 2008 Philippine National Demographic and Health Survey (NDHS).
	World Health Organization, Centers for Disease Control and Prevention (2011). Philippines Global School-based Student Health Survey 2011. Geneva, Switzerland: World Health Organization.
	Philippines Global School-based Student Health Survey, 2011
	Philippine Statistics Authority (PSA) and ICF. (2013). 2013 Philippine National Demographic and Health Survey (NDHS).
	Food and Nutrition Research Institute (2013). Burden of selected risk factors to non-communicable diseases (NCDs) among Filipino adults. Taguig, Philippines: Certification International.
	World Health Organization, Centers for Disease Control and Prevention (2015). Global School-based Student Health Survey – Philippines 2015 Fact Sheet.
	Department of Science and Technology - Food and Nutrition Research Institute (DOST-FNRI) (2016). Philippine Nutrition Facts and Figures 2015: Clinical and Health Survey.
	Philippine Statistics Authority (PSA) and ICF. (2017). 2017 Philippine National Demographic and Health Survey (NDHS).
	Philippines Expanded National Nutrition Survey, 2018–2019.
	Philippines Global School-based Student Health Survey, 2019.
	Department of Science and Technology, Food and Nutrition Research Institute (2021). Overview and Methodology: Expanded National Nutrition Survey National Dissemination Forum 2021 Survey Results.
	Philippine Statistics Authority (PSA), ICF (2023). 2022 Philippine National Demographic and Health Survey (NDHS): Final Report. (https://dhsprogram.com/pubs/pdf/FR381/FR381.pdf. Accessed: 01/27/2024).
	World Health Organization, Centers for Disease Control and Prevention (2023). Global School-based Student Health Survey – Philippines 2019 Fact Sheet. (https://extranet.who.int/ncdsmicrodata/index.php/catalog/944. Accessed: 01/28/2024).
**Singapore**	Epidemiology and Disease Control Division, Ministry of Health, Singapore (1993). National Health Survey 1992.
	Epidemiology and Disease Control Division, Ministry of Health, Singapore (1999). National Health Survey 1998.
	Epidemiology and Disease Control Division, Ministry of Health, Singapore (2005). National Health Survey 2004.
	Epidemiology and Disease Control Division, Ministry of Health, Singapore (2011). National Health Survey 2010 Singapore.
	Singapore Mental Health Study 2010 (see Subramaniam, M. et al., 2019).
	Subramaniam M, Abdin E, Vaingankar JA, Shafie S, Chua BY, Sambasivam R, Zhang YJ, Shahwan S, Chang S, Chua HC, Verma S, James L, Kwok KW, Heng D, Chong SA. Tracking the mental health of a nation: prevalence and correlates of mental disorders in the second Singapore mental health study. Epidemiol Psychiatr Sci. 2019 Apr 5;29:e29. doi: 10.1017/S2045796019000179. PMID: 30947763; PMCID: PMC8061188. [Singapore Mental Health Study 2010 and 2016].
	Epidemiology and Disease Control Division and Policy, Research & Surveillance Group, Ministry of Health and Health Promotion Board (2020). National Population Heath Survey 2017 (Household Interview). Republic of Singapore: Ministry of Health.
	Ong LT (2018). RE: NHSS 2013 data provided by Ministry of Health, Singapore. (Accessed: 02/01/2018).
	Epidemiology and Disease Control Division and Policy, Research & Surveillance Group, Ministry of Health and Health Promotion Board (2020). National Population Heath Survey 2019 (Household Interview). Republic of Singapore: Ministry of Health.
	Lee YY, Wang P, Abdin E, Chang S, Shafie S, Sambasivam R, et al. (2020). Prevalence of binge drinking and its association with mental health conditions and quality of life in Singapore, Addictive Behaviors.100:106114.
	Epidemiology and Disease Control Division and Policy, Research & Surveillance Group, Ministry of Health and Health Promotion Board (2020). National Population Heath Survey 2020 (Household Interview). Republic of Singapore: Ministry of Health.
	Epidemiology and Disease Control Division and Policy, Research & Surveillance Group, Ministry of Health and Health Promotion Board (2020). National Population Heath Survey 2021 (Household Interview). Republic of Singapore: Ministry of Health.
	Epidemiology and Disease Control Division and Policy, Research & Surveillance Group, Ministry of Health and Health Promotion Board (2022). National Population Heath Survey 2022 (Household Interview). Republic of Singapore: Ministry of Health.
	Epidemiology and Disease Control Division and Policy, Research & Surveillance Group, Ministry of Health and Health Promotion Board (2022). National Population Heath Survey 2023 (Household Interview). Republic of Singapore: Ministry of Health.
**Viet Nam**	World Health Organization (2009b). STEPwise approach to surveillance (STEPS) Survey. (http://www.who.int/chp/steps/en/. Accessed: 01/12/2018).
	Giang KB, Van Minh H, Allebeck P (2013). Alcohol consumption and household expenditure on alcohol in a rural district in Vietnam, Global Health Action.6:18937.
	Greenfield TK, Bloomfield K, Wilsnack SC (2013e). GENAHTO Project (Gender and Alcohol’s Harm to Others). (http://genahto.org/. Accessed: 07/02/2020).
	Van Bui T, Blizzard L, Ngoc Luong K, Van Truong NL, Tran BQ, Otahal P, et al. (2015). Alcohol consumption in Vietnam, and the use of ‘Standard Drinks’ to measure alcohol intake, Alcohol and Alcoholism.51 [2]: 1–10.
	World Health Organization (2015). STEPwise approach to surveillance (STEPS) Survey.
	World Health Organization (2017a). Global School-based Student Health Survey (GSHS) Viet Nam. (http://www.who.int/chp/gshs/vietnam/en/. Accessed: 06/16/2017).
	General Statistics Office, United Nations Children’s Fund (UNICEF) (2021). Survey measuring Viet Nam Sustainable Development Goal indicators on Children and Women 2020–2021, Survey Findings Report. (https://www.unicef.org/vietnam/media/9576/file/Full%20report%20-%20MICS%206.pdf. Accessed: 01/27/2024).
	World Health Organization (2024). STEPwise approach to surveillance (STEPS) Survey, 2021.

## Abbreviations

APC: Adult alcohol *per capita* consumption (yearly measure in litres pure alcohol)
ASEAN: Association of South East Asian Nations
AUD: Alcohol use disorder
CRA: Comparative Risk assessments
EAC: East African Community
EU: European Union
FAO: Food and Agriculture Organization of the United Nations
GBD: Global Burden of Disease
GISAH: Global Information System on Alcohol and Health
HED: Heavy episodic drinking
WHO: World Health Organization
